# Hypermagnesemia caused by fecal-mass obstruction in stenotic rectal cancer following preoperative administration of magnesium citrate

**DOI:** 10.1186/s40981-024-00759-4

**Published:** 2024-12-20

**Authors:** Yuri Sato, Eiji Hashiba, Yuuma Yamazaki, Koudai Kato, Hirotaka Kinoshita, Satoko Noguchi, Tomoyuki Kudo, Kazuyoshi Hirota

**Affiliations:** 1 Department of Anesthesiology, Aomori Prefectural Central Hospital, 2-1-1 Higashitsukurimichi, Aomori, 030-8553 Japan; 2 Department of Anesthesiology, Hirosaki University Graduate School of Medicine, 5 Zaifu-cho, Hirosaki, 036-8562 Japan; 3 Department of Anesthesiology, Kensei Hospital, 2-2-2 Ogimachi, Aomori, 036-8104 Japan

**Keywords:** Hypermagnesemia, Magnesium citrate, Rectal cancer

## Abstract

**Background:**

Hypermagnesemia is a rare complication, leading to fatal cardiovascular and respiratory conditions. We present severe hypermagnesemia developed in a patient with a rectal stenosis after pretreatment with oral magnesium citrate for rectosigmoid surgery.

**Case presentation:**

A 78-year-old woman demonstrated consciousness disturbance, muscle weakness, and respiratory depression requiring tracheal intubation after preparation with oral magnesium for rectosigmoid surgery. Endoscopic examination showed a rectal obstruction due to fecal impaction. General condition improved after emergency Hartmann’s surgery. Blood test revealed a remarkable increase of serum magnesium level to 17.5 mg/dL when the general condition deteriorated, which would have been responsible for her symptoms. She was discharged from the ICU after extubation on the third postoperative day with a normal magnesium level.

**Conclusions:**

Pretreatment with large doses of oral magnesium-containing bowel cleanser may cause severe hypermagnesemia in patients with colorectal stenosis.

## Background

Hypermagnesemia is a rare complication induced by various causes such as overdosages, increased absorption, and impaired excretion, and therapeutic administration of magnesium-containing drugs is one of the major causes [1]. We report a patient with rectosigmoid cancer with rectal stenosis who developed life-threatening hypermagnesemia due to obstruction of the rectum by fecal masses after preoperative treatment with an oral bowel cleanser containing magnesium citrate solution.

### Case presentation

We obtained written informed consent from the patient for the publication of this case report.

A 78-year-old female patient, height 147 cm and weight 47 kg, was scheduled for robot-assisted high-level anterior resection of the colon. She was taking amlodipine for hypertension, magnesium oxide 0.99 g, and sodium picosulfate daily for constipation. Renal function was relatively preserved, with creatinine level of 0.65 mg/dL and eGFR of 65.8 mL/min/1.73 m^2^. She had an episode which developed dysuria, abdominal pain, and nausea after ingestion of bowel cleaner containing sodium-calcium-ascorbic acid for preparing lower gastrointestinal endoscopy, and had been referred to our hospital with a diagnosis of rectosigmoid cancer. She became restless, complaining of nausea and a sense of weakness following pretreatment with 125 mL of 13.6% magnesium citrate solution and picosulfate sodium hydrate as an oral bowel cleanser 2 days before surgery in our hospital. She developed consciousness disturbance, respiratory depression, and bradycardia in the ward, prompting an in-hospital emergency call on the next day.

She had SpO_2_ of 90% under a 10 L/min reservoir mask, sinus bradycardia with a heart rate of 20–30 beats/min, decreased level of consciousness with GCS 3 points (E1V1M1), with isocoric pupils (3.0 mm) but no light reflex. Blood gas analysis showed marked respiratory acidosis: pH 7.023, PaCO_2_ 102 mmHg, PaO_2_ 129 mmHg, HCO_3_ − 25.2 mmol/L, base excess − 9.0 mmol/L, lactic acid 6.0 mmol/L. Massive regurgitation of the gastric content through the nasal airway was noted, suggesting aspiration pneumonia with carbon dioxide narcosis. The patient was admitted to the ICU with tracheal intubation. A contrast-enhanced CT scan showed a left pleural effusion and collapse of the left lower lobe of the lung, possibly due to aspiration pneumonia, as well as a stenotic lesion in the rectum and dilatation of the colon on the oral side. Head CT showed no intracranial lesions that could decrease the level of consciousness.

**Fig. 1:**
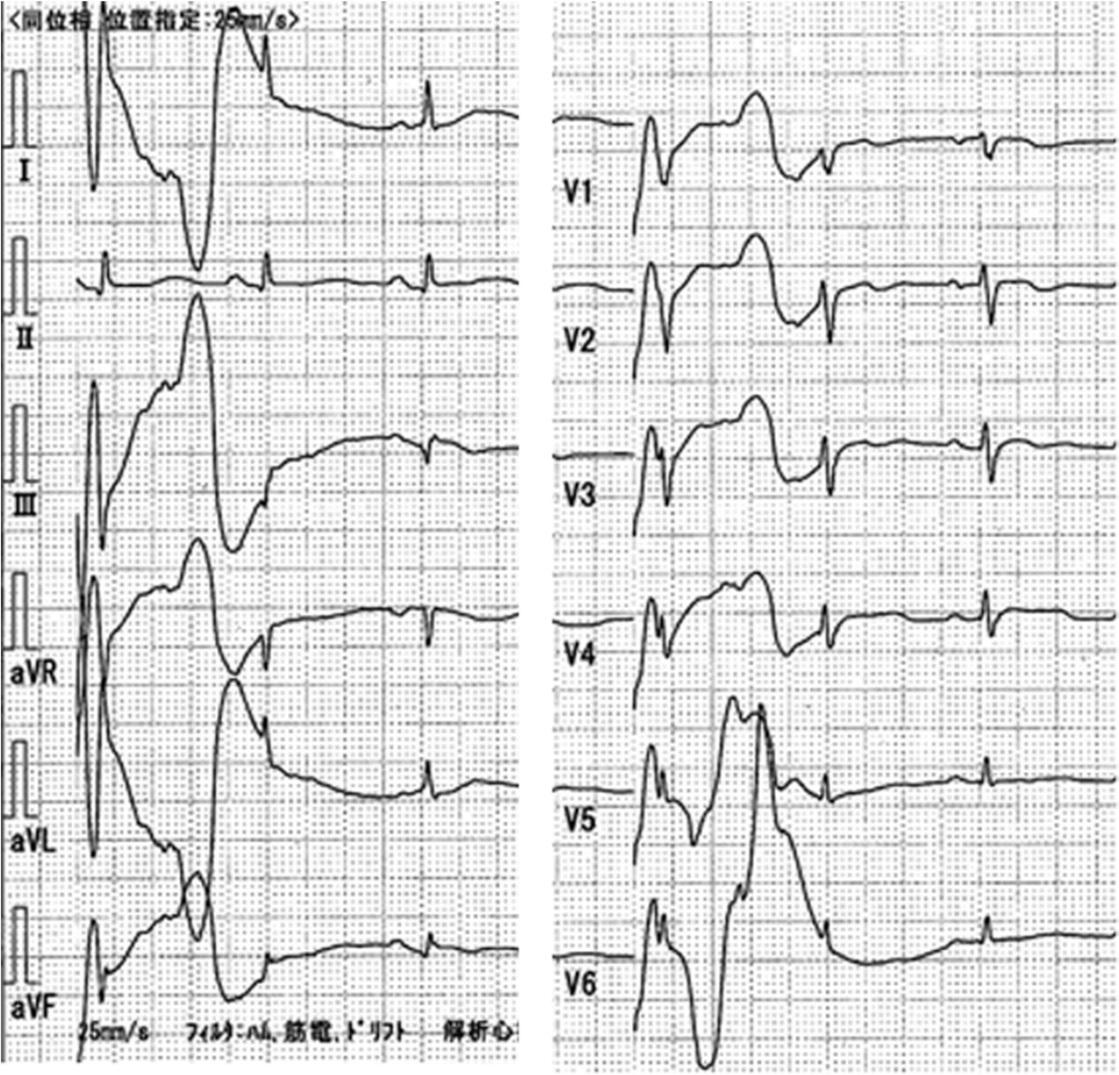
Twelve-lead ECG on ICU admission. Ventricular extrasystole, QTc 0.477, and QT prolongation were observed. Sinus bradycardia with a heart rate of 42/min is noted

The patient frequently exhibited sinus bradycardia and an escape rhythm of premature ventricular contractions, but no ischemic changes in the electrocardiogram (Fig. 1). Transthoracic echocardiography revealed no pericardial effusion and normal left ventricular wall motion. Adrenaline 0.15 μg/kg/min and dobutamine 3 μg/kg/min, along with a rapid infusion of bicarbonate Ringer’s solution, were needed to stabilize her hemodynamics. Blood tests showed normal sodium, potassium, and chlorine levels. Endoscopic examination revealed obstruction of the rectum (Rs) by fecal masses, but no evidence of ischemic or necrotic changes of the colon. As her bradycardia and hypotension improved after endoscopic release of the obstruction, a vagal reflex due to colorectal obstruction seemed to be partly involved in a series of her symptoms, and an emergency Hartmann’s operation was performed.

After surgery, continuous hemodiafiltration was introduced for acute renal failure with serum creatinine of 1.54 mg/dL and KDIGO classification Stage 1, and for metabolic acidosis. The patient’s respiratory and circulatory status remarkably improved, and the tracheal tube was removed on the day after surgery. On postoperative day 1, abnormally high magnesium level of 6.2 mg/dL was noticed, which promoted us to reexamine the stored blood samples. Magnesium levels were 2.6 and 17.5 mg/dL at the time of her admission and emergency call, respectively, suggesting that a series of this patient’s symptoms were due to hypermagnesemia probably caused by the bowel cleanser.

On the second postoperative day, urine output increased and the magnesium level decreased to 3.1 mg/dL with the use of diuretics, so continuous hemodiafiltration was terminated. The patient was discharged from the ICU to the surgical ward on the third postoperative day.

## Discussion

The pathogenesis of hypermagnesemia involves overdosages, increased absorption, and impaired excretion of magnesium [2]. In the present case, life-threatening hypermagnesemia was induced by a fecal-mass obstruction resulting from a stenotic lesion in the rectum after magnesium citrate was used in a preoperative pretreatment for rectosigmoid cancer. The increase in intestinal pressure might increase the absorption of magnesium.

Many case reports regarding hypermagnesemia exist (Table 1). Most of the reported cases of hypermagnesemia involved constipation with or without impaired renal function. Hypermagnesemia may cause smooth muscle paralysis resulting in paralytic ileus [2]. However, no case reports have mentioned the risk of obstruction with fecal masses as a mechanism of hypermagnesemia following pretreatment with oral magnesium solution in patients with stenotic intestinal lesions. The drug information for oral magnesium citrate solution warns that special caution should be exercised when administering it to patients with intestinal stenosis or severe constipation due to the risk of intestinal obstruction or perforation [3]. However, there is no warning about hypermagnesemia due to obstruction.

**Table 1:** Comparison of the present case with previously reported hypermagnesemia cases [4–10]. The present case showed the highest concentration of serum magnesium and arterial carbon dioxide, but the patient recovered. Most of the reported hypermagnesemia patients suffered from constipation or ileus. Surprisingly, renal functions of the dead cases were mildly impaired

Age/Sex	Magnesium	Daily dose of magnesium oxide	Background of gastrointestinal disease	PaCO_2_	ECG abnormality	Glasgow Coma Scale	Creatinine	Outcome
	(mg/dL)	(g)		(mmHg)		(E-V-M)	(mg/dL)	
88/F^5)^	8.3	0.66	Constipation	44.2	QT prolongation	4-1-5	5.27	Recovered
95/F^5)^	7.3	1.32	Constipation	44.2	-	4-1-5	3.56	Recovered
87/F^5)^	9.1	2	Constipation	46.2	-	4-5-6	6.4	Recovered
66/F^5)^	14.3	0.33	Constipation	45.4	Af tachycardia	1-1-5	0.66	Died
37/F^6)^	15.3	3	Constipation	40.3	PR,QT prolongation	1-1-1	2.3	Recovered
86/F^6)^	17	2	Constipation,	38.2	PR prolongation	1-1-1	1.1	Died
			Ischemic colitis?					
32/M^6)^	15.7	2	Constipation	67.6	-	1-2-5	1.3	Recovered
64/M^7)^	11	1.5	Gastric ulcer		PEA,	111	2.8	Recovered
					Junctional rhythm			
85/F^8)^	10.2	6	-		PR prolongation, bradycardia	Disturbance of consciousness	4.4	Recovered
72/F^9)^	7.4	1	Constipation		PR,QT prolongation		1.9	Died
77/F^10)^	11.9	0.99	Ileus paralytic	28.5	Atrioventricular block,	3-5-6	1.83	Recovered
					sinoatrial block			
76/F^11)^	16.6	34	Ileus	43.1	Sinus arrest	-	1.4	Recovered
		(magnesium citrate)				(Japan Coma Scale II-3)		
78/F	17.5	0.99+preoperative preparation	Rectal cancer	102	QT prolongation, bradycardia	1-1-1	1.37	Recovered
(This case)								

The Japanese Pharmacopoeia defines the upper limit of magnesium oxide administration as 2 g/day (1.2 g/day in magnesium equivalent), yet there have been cases of death even in the absence of overdosage. In the present case, the daily dosage of magnesium oxide was 0.99 mg/day, below the upper limit, and the patient’s magnesium concentration upon admission to our hospital was only slightly above the upper limit of normal (1.8 to 2.4 mg/dL). However, the large amount of magnesium citrate (17 g) administered as a pretreatment for surgery triggered lethal hypermagnesemia in the present patient due to the obstructive mechanisms mentioned above.

In addition, our patient’s renal function was mildly impaired, which may have contributed to the decreased excretion of magnesium. It is well known that a variety of nonspecific symptoms may be observed depending on the serum magnesium concentration, including nausea, vomiting, bradycardia, and hypotension at concentrations of 5 to 8 mg/dL; somnolence, loss of deep tendon reflex, and QT interval prolongation at concentrations of 9 to 12 mg/dL; coma, muscle relaxation, complete AV block, and other arrhythmias leading to cardiac arrest at concentrations of 12 mg/dL and higher [11]. Our patient had a variety of symptoms, ranging from vomiting and weakness to impaired consciousness, respiratory acidosis, and bradycardia, all of which are characteristic of hypermagnesemia. However, the diagnosis was difficult because the possibility of hypermagnesemia was not included in the differential diagnosis by the involved doctors. Fortunately, the treatments for this patient, including fluid volume infusion, resolution of the obstruction by endoscopy and surgery, and the introduction of continuous hemodiafiltration, unintentionally succeeded in decreasing the magnesium concentration.

## Conclusion

In colorectal cancer with stenosis, pretreatment with high-dose magnesium preparations prior to colonoscopy or surgery may lead to rapid absorption from the intestinal mucosa due to obstruction by fecal masses, resulting in life-threatening hypermagnesemia.

## Data Availability

Not applicable.

## References

[1] Herroeder S, Schönherr ME, De Hert SG, et al. Magnesium–essentials for anesthesiologists. Anesthesiology. 2011;114:971–93. 21364460 10.1097/ALN.0b013e318210483d

[2] Swaminathan R. Magnesium metabolism and its disorders. Clin Biochem Rev. 2003;24:47–66. 18568054 PMC1855626

[3] Magcorol ® Oral Solution 13.6% 250mL Drug Product Interview Form 9th Edition, Japan Hospital Pharmaceutical Association, 2020. https://pins.japic.or.jp/pdf/medical_interview/IF00003518.pdf. Accessed 6 Nov 2024.

[4] Yamaguchi H, Shimada H, Yoshita K, et al. Severe hypermagnesemia induced by magnesium oxide ingestion: a case series. CEN Case Rep. 2019;8:31–7. 30136128 10.1007/s13730-018-0359-5 PMC6361089

[5] Nakao S, Watanabe H, Matsuoka T. Severe hypermagnesemia caused by the long-term administration of magnesium oxide: three case reports. J Jpn Assoc Acute Med. 2010;21:365–71.

[6] So M, Ito H, Sobue K, et al. Circulatory collapse caused by unnoticed hypermagnesemia in a hospitalized patient. J Anesth. 2007;21:273–6. 17458660 10.1007/s00540-006-0492-8

[7] Hanada S, Iwamoto M, Kobayashi N, et al. Calcium-alkali syndrome due to vitamin D administration and magnesium oxide administration. Am J Kidney Dis. 2009;53:711–4. 19185403 10.1053/j.ajkd.2008.11.015

[8] Weng YM, Chen SY, Chen HC, et al. Hypermagnesemia in a constipated female. J Emerg Med. 2013;44:57–60. 10.1016/j.jemermed.2011.09.004 22244603

[9] Kanazawa A, Uchida T, Aibara K, et al. A case of acute-onset hypermagnesemia with paralytic ileus. J Jpn Soc Intensive Care Med. 2020;27:19–23.

[10] Kontani M, Hara A, Ohta S, et al. Hypermagnesemia induced by massive cathartic ingestion in an elderly woman without pre-existing renal dysfunction. Intern Med. 2005;44:448–52. 15942092 10.2169/internalmedicine.44.448

[11] Mori H, Tack J, Suzuki H. Magnesium oxide in constipation. Nutrients. 2021;13:421. 33525523 10.3390/nu13020421 PMC7911806

